# A Prospective Validation Study of Lung Cancer Gene Panel Testing Using Cytological Specimens

**DOI:** 10.3390/cancers14153784

**Published:** 2022-08-03

**Authors:** Kei Morikawa, Hirotaka Kida, Hiroshi Handa, Takeo Inoue, Hisashi Saji, Junki Koike, Seiji Nakamura, Yoshiharu Sato, Yumi Ueda, Fumihiko Suzuki, Ryo Matoba, Masamichi Mineshita

**Affiliations:** 1 Division of Respiratory Diseases, Department of Internal Medicine, St. Marianna University School of Medicine, Kawasaki 216-8513, Japan; h2kida@marianna-u.ac.jp (H.K.); hiroshihstv@marianna-u.ac.jp (H.H.); t2inoue@marianna-u.ac.jp (T.I.); m-mine@marianna-u.ac.jp (M.M.); 2 Department of Chest Surgery, St. Marianna University School of Medicine, Kawasaki 216-8513, Japan; hsaji@marianna-u.ac.jp; 3 Department of Pathological Diagnosis, St. Marianna University School of Medicine, Kawasaki 216-8513, Japan; j2koike@marianna-u.ac.jp; 4 DNA Chip Research Inc., Tokyo 105-0022, Japan; nakamura@dna-chip.co.jp (S.N.); yo-sato@dna-chip.co.jp (Y.S.); y-ueda@dna-chip.co.jp (Y.U.); f-suzuki@dna-chip.co.jp (F.S.); matoba@dna-chip.co.jp (R.M.)

**Keywords:** cytology specimens, gene panel analysis, lung cancer compact panel (LCCP), non-small cell carcinoma (NSCLC), variant allele frequency (VAF)

## Abstract

**Simple Summary:**

The gold standard for genetic panel test samples is considered to be tissue specimens. However, in clinical practice, tissue specimens are often unavailable. Therefore, using an amplicon-based high-sensitivity next-generation sequencing panel test capable of measuring eight druggable genes, we enrolled patients who underwent diagnostic procedures to evaluate analysis accuracy, nucleic acid yield, and quality using cytological specimens. Cytological specimens were suitable for both nucleic acid yield and specimen quality due to the ease of collection and processing. Cytological sample analysis detected gene mutations in 68.7% of lung adenocarcinomas, and these samples were consistent with companion diagnostic tests 99.5% of the time. Moreover, the allele frequency of gene mutations in cytological specimens showed a high correlation with tissue specimens. This is the first study to prospectively evaluate the feasibility of a lung cancer gene panel test using cytological specimens.

**Abstract:**

***Background:*** Genetic panel tests require sufficient tissue samples, and therefore, cannot always be performed. Although collecting cytological specimens is easier than tissue collection, there are no validation studies on the diagnostic accuracy of lung cancer gene panel tests using cytology samples. ***Methods:*** Using an amplicon-based high-sensitivity next-generation sequencing panel test capable of measuring eight druggable genes, we prospectively enrolled consecutive patients who underwent diagnostic procedures. We evaluated the analysis accuracy rate, nucleic acid yield, and the quality of cytological specimens under brushing, needle aspiration, and pleural effusion. We then compared these specimens with collected tissue samples. ***Results:*** In 163 prospectively enrolled cases, nucleic acid extraction and analysis accuracy was 100% in cases diagnosed with adenocarcinoma. Gene mutations were found in 68.7% of cases with 99.5% (95% CI: 98.2–99.9) concordance to companion diagnostics. The median DNA/RNA yield and DNA/RNA integrity number were 475/321 ng and 7.9/5.7, respectively. The correlation coefficient of the gene allele ratio in 64 cases compared with tissue samples was 0.711. ***Conclusion:*** The success of gene analysis using cytological specimens was high, and the yield and quality of the extracted nucleic acid were sufficient for panel analysis. Moreover, the allele frequency of gene mutations in cytological specimens showed high correlations with tissue specimens.

## 1. Introduction

Personalized medicine for lung cancer using molecular-targeted drugs and immune checkpoint inhibitors is widespread to achieve a high response and long-term prognosis [1,2,3]. To date, epidermal growth factor receptor (*EGFR*) mutation, anaplastic lymphoma kinase (*ALK*) fusion genes, c-ros oncogene1 (*ROS1*), v-raf murine sarcoma viral oncogene homolog B1 (*BRAF*), mesenchymal–epithelial transition (*MET*) exon14 skipping mutations, rearranged during transfection (*RET*) fusion genes, and their corresponding molecular-targeted drugs have been approved by the Food and Drug Administration (FDA). Furthermore, Kirsten rat sarcoma virus gene (*KRAS*) mutation, *EGFR*/human epidermal growth factor receptor 2 (*HER2*) exon20 insertion, and their corresponding molecular-targeted drugs will soon be available.

Conventionally, gene mutations have been measured by the single-plex polymerase chain reaction (PCR) method for individual gene mutations, which have high sensitivity and specificity, are relatively inexpensive, and have a short turnaround time (TAT). This method has become especially widespread, mainly for the detection of *EGFR* mutations by the cobas^®^ EGFR mutation test (Roche Molecular Systems, Pleasanton, CA, USA). However, due to the discovery of various lung cancer driver genes in the last ten years, it is not possible to test consecutive single gene mutations one after the other due to time and sample consumption constraints. In 2017, the gene panel test Oncomine Dx Target Test Multi-CDx system (Thermo Fisher Scientific, San Jose, CA, USA), which simultaneously evaluates 46 cancer-related genes, became one of the first next-generation sequencing (NGS) panels for non-small cell lung cancer testing and was approved by the FDA [4]. However, this batch test requires a sufficient amount of malignant cells in the collected tissue samples and qualified sample handling. Specimens collected by a bronchoscope often fail to produce sufficient amounts of malignant cells due to small sample sizes [5,6,7,8]. In addition, there are a certain number of cases in which a sufficient amount of tissue cannot be collected for gene batch testing, such as massive pleural effusion, cancerous lymphangiopathy with bleeding on examination, and small-sized mediastinal lymph nodes metastasis. Less invasiveness and shorter examination times may be required because of patients’ poor general condition. In the future, it is expected that the number of cases where gene mutations can be detected by liquid biopsy will increase [9,10]. However, at present, liquid panel tests might not be the first choice due to the lower sensitivity and high cost of use.

When diagnosing lung cancer in clinical practice, these aforementioned unmet needs exist for gene batch testing. Therefore, we report promising results on the development of a high-sensitivity NGS lung cancer gene panel, the lung cancer compact panel (LCCP), and its application for cytological specimens as a prospective validation study. This testing method is currently being applied for regulatory affairs with the Ministry of Health, Labor, and Welfare as a multi-companion diagnostic kit for lung cancer.

## 2. Materials and Methods

### 2.1. Aim and Study Design

The purpose of this study is to apply an LCCP to cytological specimens including brushing cytology, a needle aspiration washing solution, and pleural effusion, and evaluate the feasibility of performing gene panel tests in clinical practice. As a single-center prospective study, we evaluated the success rates of the LCCP test using a cytological specimen as a primary endpoint. Secondary endpoints were as follows: (1) Mutation call profile from cytology panel assay using LCCP and concordance with a companion diagnostic test; (2) the amount and qualitative assessment of nucleic acid (DNA/RNA) extracted from the cytological specimen; and (3) the comparison between LCCP test results using cytological specimens and using formalin-fixed paraffin-embedded (FFPE) tissues with gene allele frequency. The study was conducted in accordance with the declaration of Helsinki and approved by the Institutional Review Board of St. Marianna University School of Medicine with approval number 4814. Written informed consent was obtained from all patients.

### 2.2. Patient Selection

We enrolled consecutive patients who underwent diagnostic procedures from May 2020 to January 2022. In this study, patients with suspected lung malignancies who required pathological diagnosis and who were 20 years of age or older and had given written consent to participate in the study were eligible. Included patients were suspected of lung cell carcinoma by computed tomography (CT) or positron emission tomography–computed tomography imaging.

### 2.3. Diagnostic Procedures

For bronchoscopic examinations, endobronchial ultrasonography (EBUS) (Endoscopic Ultrasound Center; EU-ME2, Olympus, Tokyo, Japan) was routinely used in combination with a flexible thin bronchoscope, BF-P260F/P290, or normal bronchoscope, BF-1T260/1TQ290/1TH1200, with a guide sheath kit (Olympus, Tokyo, Japan). EBUS-TBNA was undertaken using a flexible fiberscope (BF-UC260F, Olympus, Tokyo, Japan), and performed 2 to 3 times with a 22-gauge needle. CT/ultrasound-guided core needle biopsy was performed 3 times with a semi-automatic aspiration device (Temno Evolution, Care Fusion Japan, Tokyo, Japan). The needle size was 20 gauge, and the length of the needle was 11 or 15 cm.

### 2.4. Sample Storage Conditions and Transport

Cytological specimens were collected in a sample container (GM-tube, Genemetrics, Osaka, Japan), which contained 2 mL of a nucleic acid stabilizer inhibiting DNase/RNase activity. Direct sample stirring and direct mixing of the washing solution were allowed up to a double dilution of the solvent. No centrifugation or freezing was required, and GM tubes were stored, refrigerated, and shipped to the inspection agency.

### 2.5. Cytological Specimen Collection Using a GM-Tube

**Figure 1:**
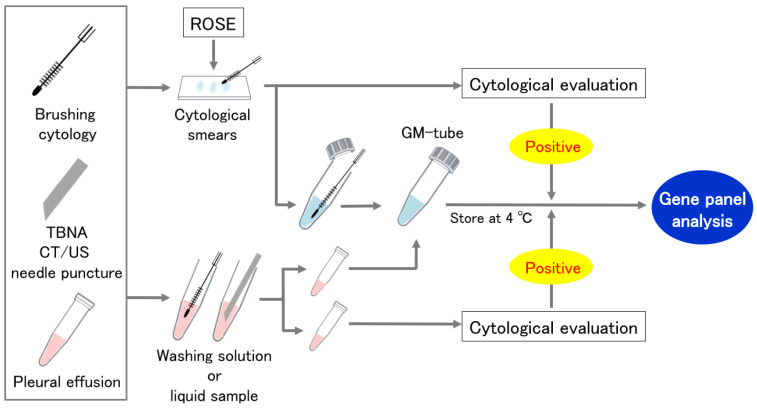
Cytological specimen collection using a GM tube. ROSE: Rapid on-site cytologic evaluation.

Cytological specimens were collected by two alternative procedures (Figure 1). In the first method, we divided the specimens between cytological smears and a GM tube for panel analysis. Cytological smears were assessed for ROSE or routine cytological evaluation for the confirmation of malignant cells. The second method divided the washing solution or liquid sample evenly into two containers, one into the GM tube and the other into the cytological evaluation. If no malignant cells were found in the paired cytological specimens, the sample was excluded from the analysis.

### 2.6. Sample Analysis

#### 2.6.1. Sample Purification, Library Preparation, and NGS Sequencing

Cytological specimens were processed with the Maxwell^®^ RSC Blood DNA Kit and Maxwell^®^ RSC simply RNA Cells Kit (Promega, WI, USA). For the FFPE specimens, DNA and RNA were purified using the Maxwell^®^ RSC DNA FFPE Kit and Maxwell^®^ RSC RNA FFPE Kit (Promega, WI, USA) according to the manufacturer’s protocol. Using the purified nucleic acid, a lung cancer compact panel (LCCP: DNA Chip Research Inc., Tokyo, Japan) NGS assay was performed as described previously [11]. The LCCP is an amplicon-based high-sensitivity NGS panel capable of measuring eight druggable genes (*EGFR*, *BRAF*, *KRAS*, *ERBB2*, *ALK*, *ROS1*, *MET*, *RET*) for lung cancer. The compact panel is characterized by highly sensitive mutation calls, with a limit of detection (LOD) for driver mutations of 0.14%, 0.20%, 0.48%, 0.24%, and 0.20% for *EGFR* exon19 deletion, L858R, T790M, *BRAF* V600E, and *KRAS* G12C, respectively. The experimental process is briefly described below. As a starting material, 5 ng of DNA (double-stranded DNA) for the DNA module and 5 ng of RNA were used for the assay of each module. Analytical validation was conducted for the assay using this input amount and proficiency was confirmed [11]. Therefore, 10 ng of dsDNA and 10 ng of RNA were set as the minimum requirements for the yield of purified nucleotides. The Qubit^TM^ fluorometer (Thermo Fisher, MA, USA) with dsDNA HS (High Sensitivity) Assay Kits and NanoDrop^®^ UV-spectrophotometry (Thermo Fisher, MA, USA) were used for the quantification of genomic DNA and total RNA, respectively. A TapeStation (Agilent, CA, USA) Genomic DNA assay was used for the assessment of purified DNA quality (DIN: DNA Integrity Number). A TapeStation (Agilent) RNA HS assay or Bioanalyzer (Agilent) was used for the assessment of purified RNA quality (RIN/eRIN: RNA Integrity Number and DV200%). For DNA assay, multiplex PCR using KOD-Plus-Neo (Toyobo, Osaka, Japan) was performed to amplify *EGFR* (exon 18–21), *BRAF* (exon 15), *KRAS* (exon 2), *ERBB2* (exon 8, 17, 20), and *MET* (near exon 14). Two DNA panels (DNA module1 and DNA module2) were designed and optimized to detect somatic mutations sensitively and quantitatively with unbiased amplification of these hotspot regions. Amplicon-based library construction was performed as described previously [11]. The library construction process is briefly described below. Forty cycles of 98 °C for 10 s and 62 °C for 30 s were performed to amplify regions on the panel of DNA module. For RNA assays, first-strand cDNA was synthesized by Revertra-ace^®^ (Toyobo, Japan), and multiplex PCR using KOD Fx Neo (Toyobo) was performed to detect *ALK*, *ROS1*, and *RET* fusion gene variants, and *MET* exon 14 skipping. Before cDNA synthesis, input RNA was mixed with a 9-base random primer (Toyobo) and incubated at 65 °C for 5 min for denaturing RNA and hybridization with the primer. The reaction mixture was incubated at 30 °C for 10 min, and then at 42 °C for 60 min in the cDNA synthesis step. Forty cycles of 98 °C for 15 s, 60 °C for 30 s, and 68 °C for 10 s, followed by extension at 68 °C for 1 min, were performed to amplify target regions. After purification with AMPure XP (Beckman Coulter Life Sciences, CA, USA), sequence libraries from these PCR products were prepared using the GenNext^®^ NGS Library Prep Kit (Toyobo). All steps were performed according to the manufacturer’s instructions. Sequence data were acquired using MiSeq (Illumina, CA, USA) for the constructed sequence library (2 × 150 bp). For data analysis, the Illumina adapter sequences were trimmed by Trimmomatic v0.33 and paired-end sequences were joined by the FLASH v1.2.11 fastq joining tool. The joined reads were mapped on the target regions of the human genome by BWA aligner v0.7.17 and the mutation variant was determined by analyzing the bam format alignment output via custom programming scripts. For the assessment of assay quality, 5000 read depths for DNA module1 and 2000 read depths for DNA module2 were set as the minimum threshold of sequencing read pairs of each amplicon region. For the RNA module, 300 sequencing reads of internal Hprt1 amplification was set as the minimum threshold. Insufficient amplification below the coverage threshold was judged as an assay failure. The analytical performance of LCCP was thoroughly validated according to the ICH guidelines (https://www.pmda.go.jp/files/000156867.pdf (accessed on 1 June 2022)).

#### 2.6.2. Pathological Diagnosis and Companion Diagnostic Test (CDx)

Cases confirmed as adenocarcinoma by histomorphological diagnosis were evaluated according to the 2015 World Health Organization (WHO) Classification of Tumors of the Lung [12]. However, when insufficient amounts of tissue were collected, we included adenocarcinomas that were determined by cytological specimens.

A genetic test that was approved by medical insurance was performed as a companion diagnostic. For cases in which sufficient sample amounts could be collected [13], samples were preferentially submitted for the Oncomine Dx Target Test Multi-CDx system, which is a gene panel test. As a single gene search, the Cobas^®^ EGFR mutation test was used to detect *EGFR* mutations and immunohistochemistry (IHC), Ventana OptiView ALK (D5F3) (Roche Molecular Systems, Pleasanton, CA, USA) [14] and the FISH, Vysis^®^ ALK Break Apart FISH probe kit (Abbott Japan LLC, Tokyo, Japan) were used for the *ALK* mutation, and Archer^®^ MET (Invitae Corp., San Francisco, CA, USA) was used for the *MET* exon 14 skipping mutation. All other rare gene mutations were confirmed by the Oncomine Dx Target Test Multi-CDx system.

#### 2.6.3. Assessment of Concordance of Variant Allele Frequency (VAF) between FFPE and Cytology

The VAF of oncogenic mutation detected from tumor samples is considered to correlate with the neoplastic cell content/cancer cell fraction. Theoretically, the VAF of the oncogenic driver mutation corresponds to half of the neoplastic cell content, assuming there is no copy number aberration and either allele harbors the mutation. The VAF is useful information to infer the neoplastic cell content and consider the relationship with drug sensitivity, because molecular target drugs tend to be more effective for tumors with a high VAF content than a low VAF content. One possible hurdle of the cytology panel assay is the dilution of the variant allele frequency via the excessive inclusion of normal cells (white blood cells, etc.). Therefore, VAF estimations from cytology samples were possibly lower than those from FFPE samples, leading to the false negative risk of driver mutation calls. To reveal the difference in neoplastic cell content between cytology and FFPE, the concordance of VAF between cytology panel and FFPE tissue panel assay was assessed. Those with sufficient pleural effusion were used as cell blocks as a substitute for tissue. Four FFPE slides were prepared per case with a 10 μm thickness, with two slides for DNA extraction and two slides for RNA extraction. For each sample, the VAF value of the primary oncogenic mutation was selected as the best indicator of the tumor cell content. The definition of oncogenic mutation is the nucleotide substitution with the COSMIC entry, and druggable mutation was assigned as the primary mutation if multiple mutations were detected from one sample. The value “0” was assigned for the discordant result with a negative call for either sample.

### 2.7. Statistical Analysis

The Clopper and Pearson exact method was used to calculate 95% confidence intervals (CIs) for the binomial proportion of concordance (https://epitools.ausvet.com.au/ciproportion (accessed on 1 June 2022)). VAF correlation between FFPE-LCCP and cytology-LCCP was calculated by the Pearson method (R version 3.6.3).

## 3. Results

### 3.1. Patient Information, Quantity and Quality Evaluation of Purified Nucleotides

**Figure 2:**
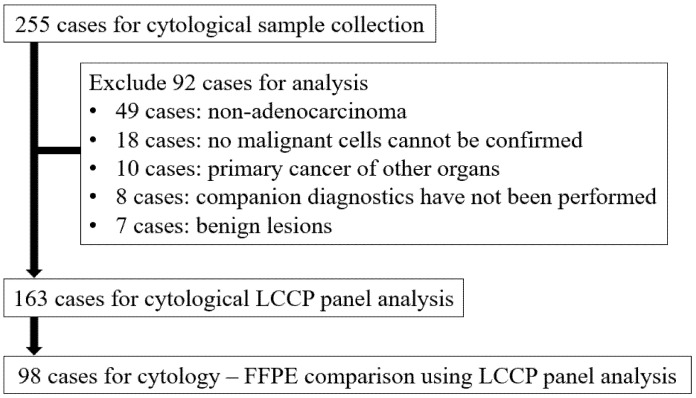
Flow diagram of sample selection. FFPE: Formalin-fixed paraffin-embedded, LCCP: Lung cancer compact panel.

The total number of cases during the enrollment period was 255, of which 92 were excluded from the analysis for the following reasons: 49 cases for non-adenocarcinoma lung cancer, 18 cases for which no malignant cells could be confirmed in paired cytological specimens, 10 cases for primary cancer of other organs, 8 cases for which companion diagnostics were not performed, and 7 cases for benign lesions. As a result, 163 cytological samples and 98 FFPE samples were analyzed by LCCP (Figure 2).

**Figure 3:**
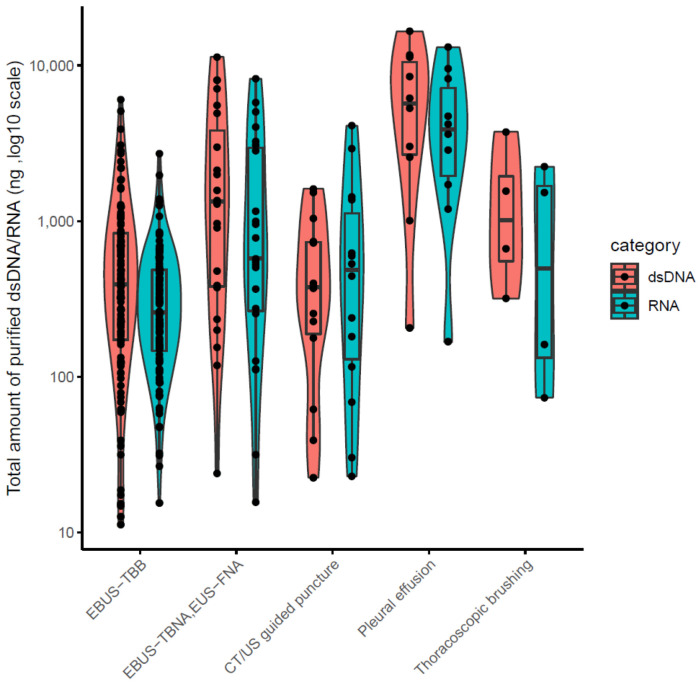
DNR/RNA yield from 163 cytology samples.

**Figure 4:**
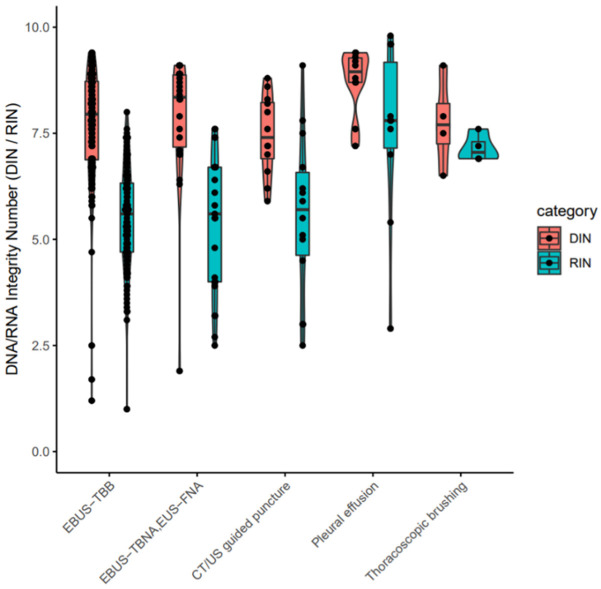
DIN/RIN values for purified nucleotides from cytology samples. EBUS-TBB: Endobronchial ultrasonography-guided transbronchial brushing. EBUS-TBNA: Endobronchial ultrasonography-guided transbronchial needle aspiration. EUS-FNA: Endoscopic ultrasound-guided fine needle aspiration. CT: Computed tomography. US: ultrasound. EBUS-TBB: Endobronchial ultrasonography-guided transbronchial brushing. EBUS-TBNA: Endobronchial ultrasonography-guided transbronchial needle aspiration. EUS-FNA: Endoscopic ultrasound-guided fine needle aspiration. CT: Computed tomography. US: Ultrasound.

The clinical stages of lung cancer were stage1/2/3/4 in 28/13/30/92 cases, respectively. The median age was 72 years (range 44–90), with 102/61 males/females (Table 1). The quality and yield of purified DNA/RNA from 163 cytology samples are summarized in Supplementary Table S1. The distribution of the purified DNA/RNA yields categorized by cytological techniques is depicted in Figure 3. For all 163 samples, purified DNA/RNA material exceeds the minimum requirement of 10 ng. The distribution of DIN/RIN for cytology samples is plotted in Figure 4. For DNA, almost all samples exceed the DIN value of 6.0 (median 7.9). For RNA, the median RIN value for 163 cytology samples was 5.7. Although the RNA quality showed different tendencies among cytological techniques (e.g., the high-quality tendency for pleural effusion and the low-quality tendency for TBNA), the quality was still sufficient for robust amplification capability of LCCP applicable to FFPE analysis. Moreover, the ratio of ss + dsDNA (double-stranded + single-stranded DNA) to dsDNA (double-stranded DNA) was estimated as an indicator of DNA damage. DNA purified from cytology samples showed a significantly lower ss + dsDNA/dsDNA ratio than that of DNA from FFPE samples (median 2.9 vs. 5.3, Welch T-test *p*-value < 0.01), representing a higher quality and the proficient quantification of DNA material purified from the cytology sample collected by a GM-tube. The ss + dsDNA/dsDNA ratio is summarized in Supplementary Table S2.

### 3.2. Assessment of Amplification Bias and Success Rate of LCCP Assay for Cytology Samples

The read depth and amplification bias estimates for 163 cytology samples and 98 FFPE samples are summarized in Supplementary Table S3. The distribution of the coefficient of variation (CV) estimated from the read depth coverage of target amplicons is plotted in Supplementary Figure S1. The median CV values for DNA module1 and DNA module2 were 0.114 and 0.308, respectively. The amplification bias estimate for all cytology samples, except only one sample, showed stable unbiased amplification below 0.3 (30% CV) for DNA module1, which covers major hotspot druggable mutations. The DNA/RNA yield in the purification step and amplification minimum read depth criteria were used for the assay success judgement in the LCCP. All 163 cytology samples exceeded the success criteria in both DNA and RNA. Thus, the LCCP assay for cytology samples showed a 100% success rate (95% confidence interval: 97.75–100%).

### 3.3. Comparison of VAF of Oncogenic Mutation Detected from Cytology-LCCP Panel Assay and FFPE-LCCP Panel Assay

**Figure 5:**
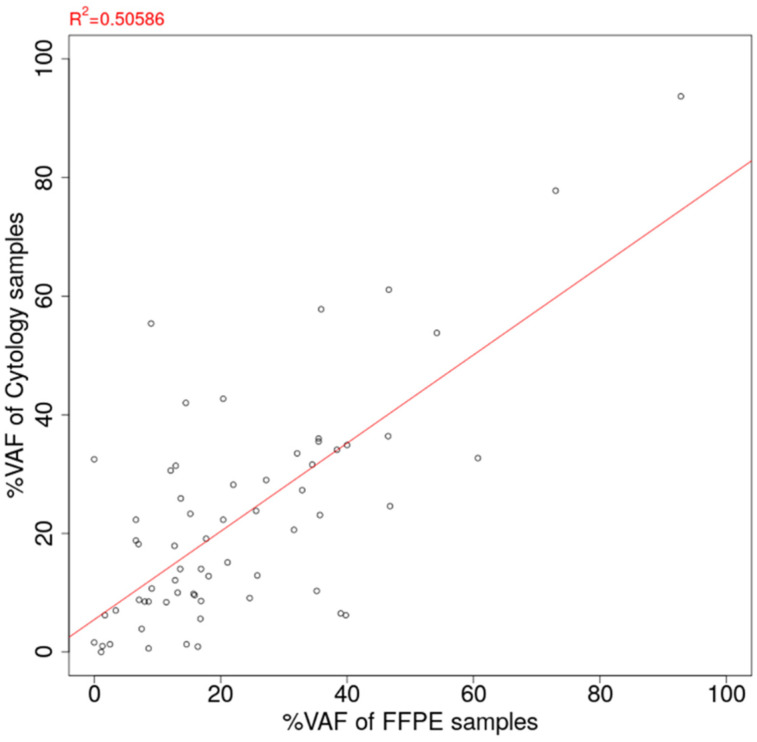
Correlation of estimated %VAF between FFPE tissue sample and cytology sample. VAF: Variant allele frequency. FFPE: Formalin-fixed paraffin-embedded.

Among the 98 cytology-FFPE matched pair samples, oncogenic mutations were detected for 64 pairs (65.3%). The VAF estimation of detected mutations for 163 cytology samples and 98 FFPE samples is summarized in Supplementary Table S4. The scatter plot of estimated VAF values is shown in Figure 5. The correlation coefficient of VAF between the cytology panel and the FFPE panel was estimated to be 0.711, showing the good agreement between the cytology panel and the FFPE tissue sample. On the contrary, there were substantial cases of greater VAF estimation in cytology than in FFPE (29/64 cases, 45.3%).

### 3.4. Mutation Call Profile from Cytology LCCP and Concordance with Companion Diagnostic Test

**Figure 6:**
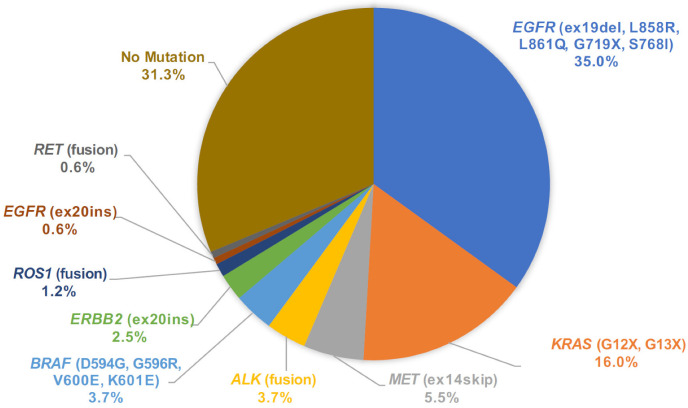
Pie chart of mutation call variants detected by LCCP assay of cytology samples. LCCP: Lung cancer compact panel.

From the mutation call results of 163 cytology samples, we assigned the primary driver mutation for each sample and summarized the driver mutation frequency in the population (Figure 6). One sample harbored dual driver co-mutations (*BRAF* V600E and *EGFR* L858R) and the *BRAF* category was assigned in this rare co-mutation case [15]. In the whole dataset of cytology LCCP, driver mutations were detected in 112 out of 163 cases (68.7%). To assess the accuracy of the mutation call from the cytology panel, *EGFR* druggable mutations detected by the companion diagnostic test were compared with the LCCP assay results. For the assay of the cytology, the *EGFR* mutation call made by LCCP in 151 cases out of 152 cases (151/152 = 99.3%) agrees with the results of the companion diagnostic mutation call. The sensitivity, specificity, positive predictive value (PPV), and negative predictive value (NPV) were estimated as 100%, 98.9%, 98.3%, and 100%, respectively. One discordant result (CP-SM-234) was the negative call by the cobas^®^ mutation detection kit v2.0 and an *EGFR* positive call by LCCP. The case was revealed to harbor dual-compound mutations of *EGFR* L858R and *EGFR* K861I. Such a compound mutation of this proximal position is difficult to detect using a probe-based PCR assay, as described in the package insert of cobas as the assay limitation. The overall sensitivity for all druggable mutations was estimated to be 98.7% (75/76 cases). In one false-negative case of cytology LCCP (CP-SM-065), *ALK* fusion was detected by Vysis^®^ ALK Break Apart FISH. To analyze the reason behind the disagreement, the residual RNA material was applied to another analytical system called the NOIR-SS molecular barcoding anchored-PCR fusion analysis system [16,17,18]. As a result, the *CLIP1-ALK* novel variant subtype of *ALK* fusion was detected by the NOIR-SS analysis [19,20]. The detection of such a rare variant subtype with a novel 5′ partner was not included in the panel design of LCCP. This type of false negative is the common limitation of the amplicon-based panel assay. For the five approved CDx mutations, *EGFR*, *ALK* fusion, *ROS1* fusion, *MET* exon14 skipping, and *BRAF* V600E, the overall concordance of mutation calls between LCCP-cytology and CDx-tissue was 99.5% (95%CI: 98.2–99.9%), suggesting the non-inferiority of cytology analysis against the tissue-based standard-of-care CDx analysis (Table 2).

## 4. Discussion

This is the first study to prospectively evaluate the feasibility of a lung cancer gene panel test in cytological specimens. We evaluated cytological specimens, not only in terms of the gene mutation analysis success rate, but also nucleic acid yield and quality with tissue sample comparisons.

First, the sample collection method does not require centrifugation or freezing and is extremely simple when compared with the handling of tissue samples for gene panel testing. However, to confirm whether the GM tube contains malignant cells, it is necessary to perform ROSE together or divide the collected sample into two separate containers, then confirm the presence of malignant cells for pairing the samples with sequential pathological evaluation. In particular, if malignant cells can be confirmed with the ROSE method, it is possible to ship the sample on the day of examination, which greatly contributes to shortening the turn-around time. On the other hand, the Oncomine Dx target test [4], which is widely used in tissue gene panel tests, takes one week from the examination date to the shipment of the sample, and the inspection results take another two weeks after sample submission.

This prospective study revealed a high success rate and correct diagnosis rate for genetic panel tests using cytological specimens. These results were supported by sufficient nucleic acid yield and high nucleic acid quality. In tissue biopsy, a nucleic acid yield of 10 ng is a prerequisite for panel examination [4], but in most cases, nucleic acid yield well exceeded the prerequisite for cytological specimens. On the contrary, in some cases, cytological specimens had a sufficient nucleic acid yield compared to tissue specimens. It is assumed that nucleic acid deterioration and fragmentation often occur in tissue specimens during the formalin fixation process, resulting in a decrease in nucleic acid yield [19], while in the cytological sample, the collected intracellular nucleic acid is stored safely in a GM tube, which resulted in little loss for nucleic acid yield.

In this study, sufficient and high-quality nucleic acid yields were obtained regardless of the test method. However, the fact that the yield of RNA is slightly lower than that of DNA or the RIN value is lower than that of DIN is presumed to be due to the rapid degradation of RNA [20,21]. In particular, the high nucleic acid yield of pleural effusion is presumed to be due to the contamination of various cells other than cancer cells, but it is a fact that the quality of nucleic acid is also extremely high [22,23].

The positive predictive value of gene mutations between LCCP and health insurance gene analysis was extremely high. In only one case was the *ALK* fusion gene not detected by LCCP due to the rare *ALK* fusion mutation *CLIP1-ALK* [24,25], which was not recognized as a detectable variant type. Hence, for *ALK*-fusion NSCLC, the use of immunohistochemistry (IHC) is also useful as a screening method for detecting *ALK* mutations. In addition, the results from LCCP gene mutation using cytological samples and the tissue samples were all in agreement.

It is also noteworthy that this study revealed that there is a high correlation between the frequency of gene alleles detected in cytopathological specimens and tissue specimens. In particular, when the tumor content was low in tissue samples, that is, in cases where the tissue gene panel test was unsuitable, a higher gene allele ratio was detected in the cytological sample, and it was considered that the cytological sample was superior. As the number of gene batch tests increases, there have been reports on the expression of multiple gene mutations. Therefore, information on gene allele frequency may be important for treatment selection and the prediction of the effects of treatment [15,26,27].

At present, a less invasive and highly accurate inspection method is required. It goes without saying that the least invasive gene search is liquid biopsy [9]. However, since bronchoscopy is often used for the definitive diagnosis of lung cancer [28,29], we can often experience adverse events such as bleeding when collecting samples. The ability to perform gene panel tests on cytological specimens can greatly contribute to ensuring patient safety [30]. In addition, it is problematic that many cases cannot be submitted or that test results fail for Oncomine Dx target testing in daily medical care [31]. With the spread of gene panel tests using cytological specimens, an increase in gene mutations, including rare genes, will be easier to detect. As a result, the use of molecular-targeted drugs will improve the prognosis of many patients [1].

The methodological limitations of this study are that, firstly, it is a prospective study at a single institution. Currently, we are conducting a prospective verification study, which is underway at multiple domestic institutions. Secondly, even though it is a gene batch test, NGS analysis is limited to eight druggable genes. However, LCCP has scalability in mutation measurement items, and is an analysis method that can add more types and variants of gene mutations in the future. Third, the method of collecting cytological specimens will need to be standardized at each facility in line with daily medical care. The standardization of sample collection procedures is also underway for verification in the ongoing multi-center study.

## 5. Conclusions

The success rate of gene analysis using cytological specimens was high, and the yield and quality of the extracted nucleic acid were also sufficient for panel analysis. Moreover, the allele frequency of gene mutations in cytological specimens showed a correlation with tissue specimens.

## Figures and Tables

**Table 1:** Patient characteristics. EBUS-TBB: Endobronchial ultrasonography-guided transbronchial brushing. EBUS-TBNA: Endobronchial ultrasonography-guided transbronchial needle aspiration. EUS-FNA: Endoscopic ultrasound-guided fine needle aspiration. CT: Computed tomography. US: Ultrasound.

Patient Characteristic	*n* = 163
Male/female	102/61
Median age, years(range)	72(44–90)
Clinical stage(I/II/III/IV)	28/13/30/92
**Diagnostic** **procedure**	
EBUS-TBB	112
EBUS-TBNA, EUS-FNA	23
CT/US guided puncture	14
Pleural effusion	10
Thoracoscopic brushing	4

**Table 2:** Concordance of mutation call by CDx-tissue and LCCP-cytology. CDx: Companion diagnostic test. LCCP: Lung cancer compact panel. PPV: Positive predictive value. NPV: Negative predictive value.

			LCCP					
		Gene	Positive	Negative	Sensitivity	Specificity	PPV	NPV
**CDx**	Positive	*EGFR*	57	0	1.000 (0.949–1.000)	-	0.983 (0.908–1.000)	-
	Negative		1	94	-	0.989 (0.943–1.000)	-	1.000 (0.969–1.000)
	Positive	*ALK*	6	1	0.857 (0.421–0.996)	-	1.000 (0.607–1.000)	-
	Negative		0	82	-	1.000 (0.964–1.000)	-	0.988 (0.935–1.000)
	Positive	*BRAF* V600E	4	0	1.000 (0.473–1.000)	-	1.000 (0.473–1.000)	-
	Negative		0	48	-	1.000 (0.939–1.000)	-	1.000 (0.939–1.000)
	Positive	*ROS1*	2	0	1.000 (0.224–1.000)	-	1.000 (0.224–1.000)	-
	Negative		0	53	-	1.000 (0.945–1.000)	-	1.000 (0.945–1.000)
	Positive	*MET*	6	0	1.000 (0.607–1.000)	-	1.000 (0.607–1.000)	-
	Negative		0	53	-	1.000 (0.945–1.000)	-	1.000 (0.945–1.000)
	Positive	ALL 5 Genes	75	1	0.987 (0.929–1.000)	-	0.987 (0.929–1.000)	-
	Negative		1	330	-	0.997 (0.983–1.000)	-	0.997 (0.983–1.000)

## Data Availability

The gene analysis data have been registered in the DDBJ Sequence Read Archive (DRA). BioSample accession(s): SAMD00502993-SAMD00503155 Temporary Submission ID: SSUB022187 Release: Hold (not viewable until the release of linked data).

## Supplementary Materials

The following supporting information can be downloaded at: https://www.mdpi.com/article/10.3390/cancers14153784/s1, Table S1: Quality assessment result of purified nucleotide; Table S2: ss+dsDNA/dsDNA ratio for purified nucleotides; Table S3: Read coverage depth of amplicon regions for cytology Compact Panel assay; Table S4: VAFs (Variant Allele Frequency) of driver mutation calls for Cytology and FFPE samples.
